# Pediatric epilepsy surgery from 2000 to 2018: Changes in referral and surgical volumes, patient characteristics, genetic testing, and post-surgical outcomes

**DOI:** 10.1111/epi.17670

**Published:** 2023-07-21

**Authors:** Maria H Eriksson, Kirstie J Whitaker, John Booth, Rory J Piper, Aswin Chari, Patricia Martin Sanfilippo, Ana Perez Caballero, Lara Menzies, Amy McTague, Sophie Adler, Konrad Wagstyl, Martin M Tisdall, J Helen Cross, Torsten Baldeweg

**Affiliations:** 1 Developmental Neurosciences Research and Teaching Department, UCL Great Ormond Street Institute of Child Health, London, UK; 2 Department of Neuropsychology, Great Ormond Street Hospital NHS Trust, London, UK; 3 The Alan Turing Institute, London, UK; 4 Department of Neurology, Great Ormond Street Hospital NHS Trust, London, UK; 5 Digital Research Environment, Great Ormond Street Hospital NHS Trust, London, UK; 6 Department of Neurosurgery, Great Ormond Street Hospital NHS Trust, London, UK; 7 North Thames Genomic Laboratory Hub, Great Ormond Street Hospital NHS Trust, London, UK; 8 Department of Clinical Genetics, Great Ormond Street Hospital NHS Trust, London, UK; 9 Imaging Neuroscience, UCL Queen Square Institute of Neurology, London, UK; 10 Young Epilepsy, Lingfield, UK

**Keywords:** Epilepsy surgery, Pediatric, Outcome, Antiseizure medication, Trends

## Abstract

**Objective:**

Neurosurgery is a safe and effective form of treatment for select children with drug-resistant epilepsy. Still, there is concern that it remains underutilized, and that seizure freedom rates have not improved over time. We investigated referral and surgical practices, patient characteristics, and post-operative outcomes over the past two decades.

**Methods:**

We performed a retrospective cohort study of children referred for epilepsy surgery at a tertiary center between 2000 and 2018. We extracted information from medical records and analyzed temporal trends using regression analyses.

**Results:**

1,443 children were evaluated for surgery. Of these, 859 (402 females) underwent surgical resection or disconnection at a median age of 8.5 years (IQR=4.6-13.4). Excluding palliative procedures, 67% of patients were seizure-free and 15% were on no antiseizure medication at one-year follow-up. There was an annual increase in the number of referrals (7% [95% CI=5.3-8.6], p<0.001) and surgeries (4% [95% CI=2.9-5.6], p<0.001) over time. Duration of epilepsy and total number of different antiseizure medications trialed from epilepsy onset to surgery were, however, unchanged, and continued to exceed guidelines. Seizure freedom rates were overall also unchanged but showed improvement (OR 1.09 [95% CI=1.01-1.18], p=0.027) after adjustment for an observed increase in complex cases. Children who underwent surgery more recently were more likely to be off antiseizure medications post-operatively (OR 1.04 [95% CI=1.01-1.08], p=0.013). There was a 17% annual increase ([95% CI=8.4-28.4], p<0.001) in children identified to have a genetic cause of epilepsy, which was associated with poor outcome.

## Introduction

Epilepsy surgery is widely regarded as a safe and effective form of treatment for select children with drug-resistant epilepsy. Approximately 65% of children are seizure-free one year after surgery, and 60% of children maintain their seizure freedom five years after surgery.^1^ Importantly, a significant proportion of children who undergo epilepsy surgery also experience an improvement in cognition and a reduced need for antiseizure medication (ASM).^2^

The past two decades have seen growing calls for increased and earlier access to epilepsy surgery in children.^3,4^ Randomized controlled trials conducted in both pediatric^5^ and adult^6^ patients have unequivocally demonstrated the superiority of epilepsy surgery over continued medical treatment in patients deemed to be candidates for surgery. Advancements in the pre-surgical assessment have, furthermore, allowed formerly ineligible patients to be considered for surgery.^7^ Finally, shorter duration of epilepsy prior to surgery has repeatedly been associated with more favorable seizure outcome and successful withdrawal of ASM.^8,9^

Despite this, there is concern as to whether epilepsy surgery remains an underutilized form of treatment.^4,10,11^ There is also concern as to whether progress with regard to seizure outcome has been made in more recent years. To date, few studies have examined trends in seizure freedom rates after epilepsy surgery in children, and they have reported conflicting findings of both improvements^12,13^ and no change.^8,14^ One hypothesis is that an increase in case complexity over time may have counteracted an improvement in seizure freedom rates; however, this remains to be demonstrated in a pediatric cohort. More recently, there has also been an increased focus on the role of genetic testing in pre-surgical planning^15^, though little is known about its significance in relation to seizure outcome.

To address these outstanding questions, we aimed to investigate changes in 1) referral and surgical volumes, 2) disease duration and total number of different ASMs trialed from epilepsy onset to pre-surgical evaluation, 3) patient characteristics, 4) the use of genetic testing, and 5) seizure outcome and post-operative ASM status following pediatric epilepsy surgery over the past two decades.

## Methods

### Patient cohort

We retrospectively reviewed medical records for all children referred and evaluated for epilepsy surgery at Great Ormond Street Hospital (GOSH; London, UK) from 1 January 2000 through 31 December 2018. We chose the year 2000 as the starting point for data collection to ensure adequate availability of high-quality data.

We included patients who underwent surgical resection or disconnection. We excluded patients who underwent surgical neuromodulation (as deep brain stimulation and responsive neurostimulation are not approved or commissioned procedures for children with epilepsy in the UK) and thermocoagulation (as this is primarily used as a prognostic test rather than definitive treatment in the UK^16^). If patients had undergone multiple resective and/or disconnective surgeries over the course of the study period, we included only their first surgery.

### Data retrieval and classification

We extracted the following information from medical records: patient demographics, epilepsy characteristics, pre-operative MRI findings, pre-operative ASM (both the total number of different ASMs that the patient had trialed from the time of their epilepsy onset to the time of their pre-operative evaluation, and the number of ASMs that the patient was receiving at the time of their pre-operative evaluation), surgery details, genetic results, histopathology diagnoses, and post-operative outcomes. We classified patients as either seizure-free (including no auras) or not seizure-free, and recorded if patients were on, weaning or off ASMs at one-year follow-up.

A complete list of variables extracted and information about how we classified these data can be found in Supplementary Material (p. 2-5).

### Statistical analysis

We calculated the descriptive statistics for the cohort using mean with standard deviation, median with interquartile range (IQR), and count with proportion, as appropriate.

We performed univariable negative binomial regression analyses to investigate if the annual number of referrals, surgeries, surgery types, pre-operative MRI findings, genetic diagnoses, and histopathology diagnoses changed over time, from 2000 to 2018. We chose negative binomial regression analyses due to overdispersion observed in Poisson regression analyses. Referral count, procedure count, or diagnosis count were the dependent variables and calendar year was the key independent variable. We checked for excess zeros in the data, to determine if a zero-truncated model would be more appropriate. We further checked model residuals for signs of autocorrelation and tested for this using the Breusch-Godfrey test. We performed an exponential transformation on the regression coefficients to calculate a percentage change in the count number. We presented these with 95% confidence intervals (CIs). We additionally performed univariable negative binomial regression analyses to investigate if the proportion of different surgery types, pre-operative MRI findings, genetic diagnoses, and histopathology diagnoses changed over time by including the total number of cases each year (logged) as an offset.

We investigated associations between epilepsy characteristics (age of epilepsy onset, age at surgery, duration of epilepsy, and total number of different ASMs trialed from epilepsy onset to pre-surgical evaluation) and date of surgery, which was transformed into a numerical variable for analysis purposes, using Spearman's rank-order correlation. We explored potential differences in duration of epilepsy and total number of ASMs trialed from epilepsy onset to pre-surgical evaluation between genetic groups (“No test”, “Positive finding”, and “Negative finding”) using Kruskal-Wallis test by ranks.

We performed a univariable logistic regression analysis to investigate if date of surgery predicted seizure outcome at one-year follow-up. We repeated this for palliative and non-palliative procedures, respectively. We then examined if seizure freedom rates improved when complex cases were excluded. We considered patients with an inherent lower a priori probability of achieving seizure freedom as complex, including those who were MRI negative, had bilateral or non-focal MRI abnormalities, underwent corpus callosotomy, disconnective surgery, or surgery involving multiple approaches (e.g. lobectomy and lesionectomy), had histopathology diagnoses such as non-specific findings, tuberous sclerosis, and focal cortical dysplasia type I, and/or had a genetic finding. We also performed multivariable logistic regression analyses to investigate if there was an interaction effect between date of surgery and surgery type, pre-operative MRI findings, and histopathology diagnosis, on seizure outcome. Finally, we performed a univariable ordinal logistic regression analysis to investigate if date of surgery predicted ASM status at one-year follow-up. We reported odds ratios (ORs) and corresponding 95% CIs for all logistic regression analyses.

We performed all statistical analysis and visualizations in R version 3.6.3. All tests were two-tailed, and we set the threshold for significance a priori at p<0.050. We performed correction for multiple comparison where appropriate using the Holm method.

## Results

### Referral and surgical volumes

In total, 1,443 children were referred and evaluated for epilepsy surgery at GOSH between 2000 and 2018. Of these, 859 (60%) went on to have first-time surgical resection or disconnection. A flowchart of patient inclusion can be viewed in Supplementary Figure 1.

Demographic information for the included patients is displayed in Table 1. Patients were referred from throughout the UK (Supplementary Figure 2).

**Figure 1:**
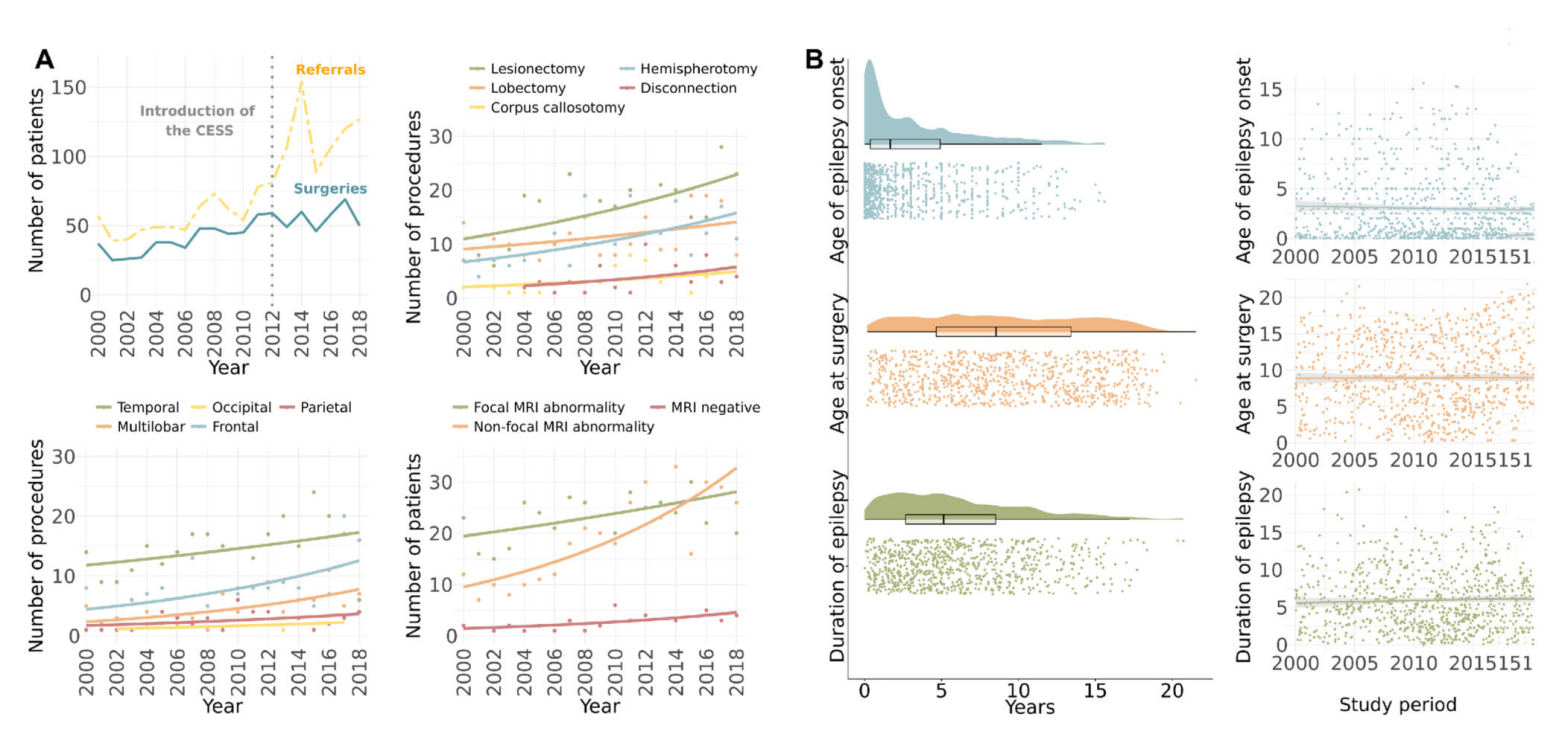
Changes in surgery practices and epilepsy characteristics between 2000 and 2018. (A) *Referrals and surgeries.* Top left: There was an increase in the annual number of children referred and evaluated for surgery, the annual number of children who underwent surgery, and the annual number of children who were reviewed but rejected for surgery, between 2000 and 2018. Top right: Changes in the type of surgery performed. Multiple subpial transection procedures were excluded from analysis due to small sample (N=5). Bottom left: Changes in lobe operated on. Bottom right: Changes in pre-operative MRI findings. (B) *Epilepsy characteristics*. Left: Raincloud plots^32^ show the raw data, box plots, and density functions for age of epilepsy onset, age at surgery, and duration of epilepsy across the cohort. Age of epilepsy onset was heavily skewed, with most children receiving a diagnosis of epilepsy before 5 years of age. Age at surgery was, in contrast, evenly distributed across childhood. Right: There was no change in age of epilepsy onset, age at surgery, or duration of epilepsy from 2000 to 2018. Abbreviations: CESS = Children’s Epilepsy Surgery Service.

Between 2000 and 2018, there was a 7% annual increase in the number of referrals (95% CI=5.3-8.6, p<0.001) and a 4% annual increase in the number of surgeries (95% CI=2.9-5.6, p<0.001) (Figure 1A). The proportion of referred and evaluated children proceeding to surgery decreased by 2% each year (95% CI=-3.9--0.8, p=0.003).

### Surgery types

Lesionectomy (36%) and lobectomy (25%) were the most commonly performed procedures (Table 1). The majority of surgeries involved the temporal lobes (32%), followed by the frontal lobes (17%). Few surgeries involved the parietal, occipital or insular lobes (each contributing <5% of all procedures).

There was a 9% annual increase in the proportion of disconnective surgeries (95% CI=2.3-17.5, p=0.040). There was no change in the proportion of surgeries by lobe operated on (all p>0.380) (Supplementary Table 1).

### Epilepsy characteristics

**Figure 2:**
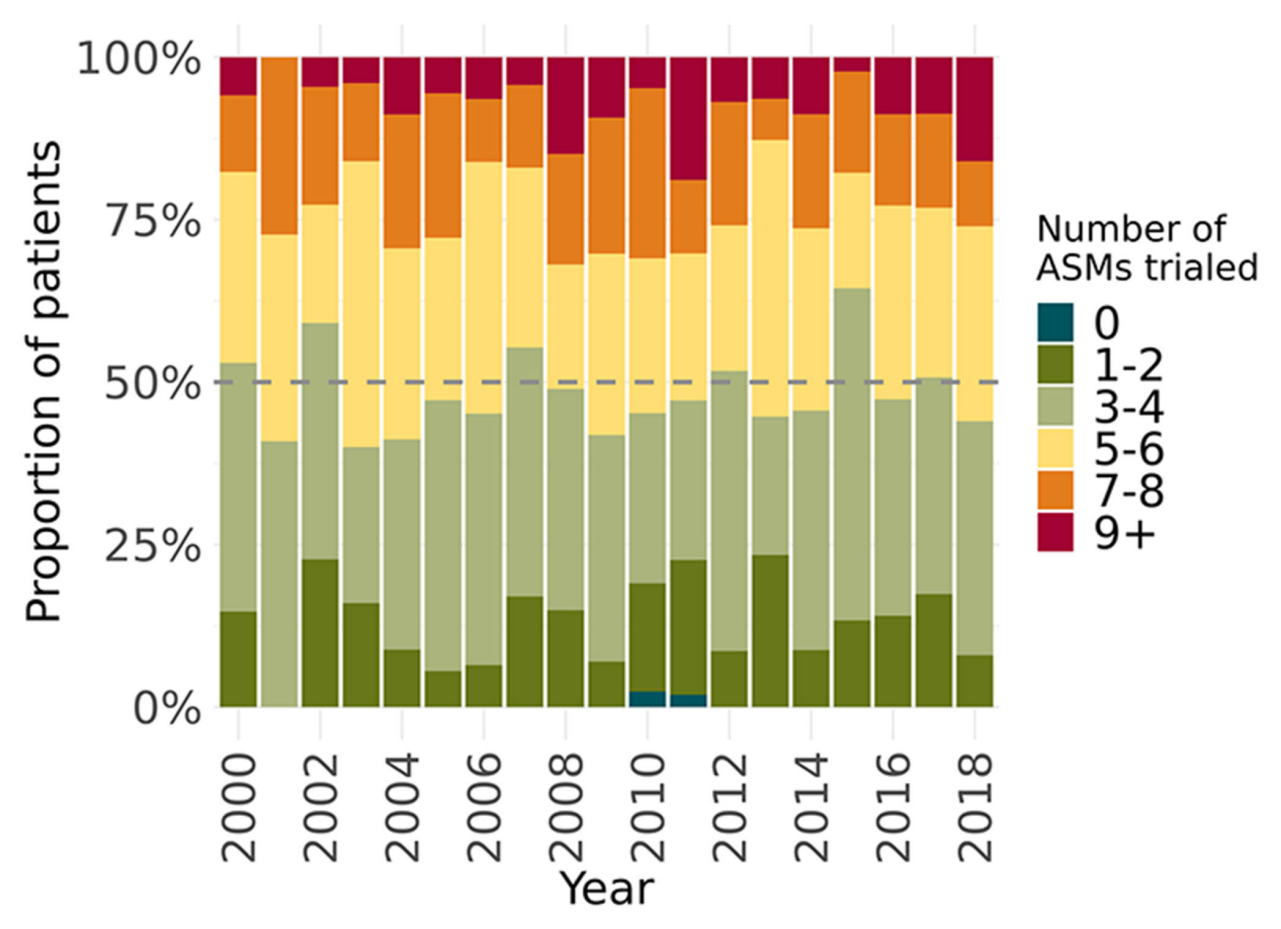
Changes in number of antiseizure medications trialed prior to surgery. There was no change in the proportion of children who had been trialed on a high number of ASM prior to surgery. Abbreviations: ASM = Antiseizure medication.

Age of epilepsy onset, age at surgery, and duration of epilepsy at time of surgery are displayed in Figure 1B. Overall, there were no changes in these characteristics over time (Figure 1B) (Supplementary Table 2). The mean number of different ASMs trialed from time of epilepsy onset to time of pre-surgical evaluation was 5.0 (SD=2.5), and this did not change over time (r=0.15, p=0.532). Each year, approximately 50% of patients had been trialed on a total of 5 or more different ASMs, and nearly 25% had been trialed on a total of 7 or more since their epilepsy onset (Figure 2).

### Pre-operative MRI findings

The majority of patients (94%) had an abnormal MRI at time of surgery (Table 1). Of these patients, 44 had previously been reported as MRI negative. The histopathology findings of patients who were MRI negative at time of surgery, as well as patients who were previously reported as MRI negative but MRI positive at time of surgery, are described in Supplementary Table 3.

Focal (52%) MRI abnormalities were more common than multifocal (11%) and diffuse (31%) MRI abnormalities amongst patients who were MRI positive at time of surgery.

There was a 3% annual increase in the proportion of non-focal (diffuse and multifocal) MRI abnormalities (95% CI=1.0-5.0, p=0.015). This was accompanied by a 2% annual decrease in the proportion of focal MRI abnormalities (95% CI=-3.9--0.4, p=0.028) (Figure 1A).

### Genetic findings

In total, 125 patients underwent genetic testing. Of these, 63 (50%) had an abnormal finding (i.e. genetic variant of possible diagnostic significance). A pathogenic, or likely pathogenic, variant was found in 34 of these patients: 22 patients had a variant caused by a single nucleotide variation (SNV) (Table 2) and 12 patients had a variant caused by a copy number variation (CNV) (Supplementary Table 4). A significant proportion (32%) of patients with a genetic cause of epilepsy did not undergo genetic testing, or obtain their genetic result, until after surgery.

The seizure freedom rate of patients with a pathogenic, or likely pathogenic, SNV was 25%, and the seizure freedom rate of those with a CNV was 33%. Patients with a (likely) benign SNV had a seizure freedom rate of 30%, while patients with no variant identified had a seizure freedom rate of 51%. There was no difference in duration of epilepsy between patients based on their genetic findings (H(2)=2.2, p=0.335). There was, however, a difference in the total number of different ASMs trialed from epilepsy onset to pre-surgical evaluation by genetic group (H(2)=8.3, p=0.016), whereby patients with a negative genetic test (mean=5.8, SD=2.9) had been trialed on more ASMs than patients who had not undergone genetic testing (mean=4.9, SD=2.4, p=0.015).

There was a 20% annual increase in the proportion of children who underwent genetic testing (95% CI=14.6-25.9, p<0.001). Correspondingly, there was a 17% annual increase in the proportion of children with a genetic diagnosis (95% CI=8.4-28.4, p<0.001) (Supplementary Figure 3).

### Histopathology diagnoses

Histopathology results are displayed in Table 1. Low-grade epilepsy-associated tumor (18%), focal cortical dysplasia type II (14%), and mesial temporal sclerosis (8%) represented the most frequent diagnoses. Conversely, non-low-grade epilepsy-associated tumor, focal cortical dysplasia not otherwise specified, and mild malformation of cortical development represented the least frequent (each contributing <1% of all diagnoses).

There was an 18% annual increase in the proportion of patients with non-specific epilepsy-associated changes (95% CI=7.7-31.6, p=0.005) (Supplementary Table 1).

### Seizure outcome

Seizure freedom rates are reported in Supplementary Table 5. Excluding palliative procedures of corpus callosotomy and multiple subpial transections, 67% of patients were seizure-free one year after surgery.

**Figure 3:**
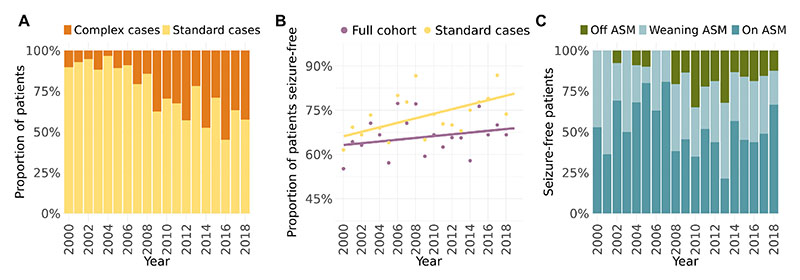
Changes in seizure outcome and post-operative antiseizure medication status between 2000 and 2018. (A) There was an increase in the proportion of complex cases over time. (B) When considering the cohort as a whole, there was no temporal change in the probability of achieving seizure freedom (purple line). However, after excluding complex cases, patients were more likely to become seizure-free over time (yellow line). Lines have been fitted to individual level data. Points represent the proportion of patients seizure-free in a given year. (C) Patients were over time more likely to be weaning or on no ASM after surgery. Abbreviations: ASM = Antiseizure medication.

Across the study period, seizure freedom rates remained stable (OR 1.01 [95% CI=0.98-1.03], p=0.678). This was also true for both palliative (OR 0.99 [95% CI=0.82-1.22], p=0.927) and non-palliative procedures (OR 1.01 [95% CI=0.98-1.04], p=0.560). Seizure freedom rates did, however, improve over time (OR 1.09 [95% CI=1.01-1.18], p=0.027) when we accounted for the increase in the proportion of complex cases (Figure 3A and 3B).

We also found that patients with a diagnosis of malformation of cortical development-other were over time less likely to achieve seizure freedom (OR 0.78 [95% CI=0.59-0.97], p=0.040) (Supplementary Table 6 and Supplementary Figure 4). This trend could be explained by changes in the frequency of diagnoses within the malformation of cortical development-other category (Supplementary Figure 5). There was no interaction between date of surgery and pre-operative MRI findings, or date of surgery and surgery type, on seizure outcome (Supplementary Tables 7 and 8).

### Post-operative antiseizure medication withdrawal

**Figure 4:**
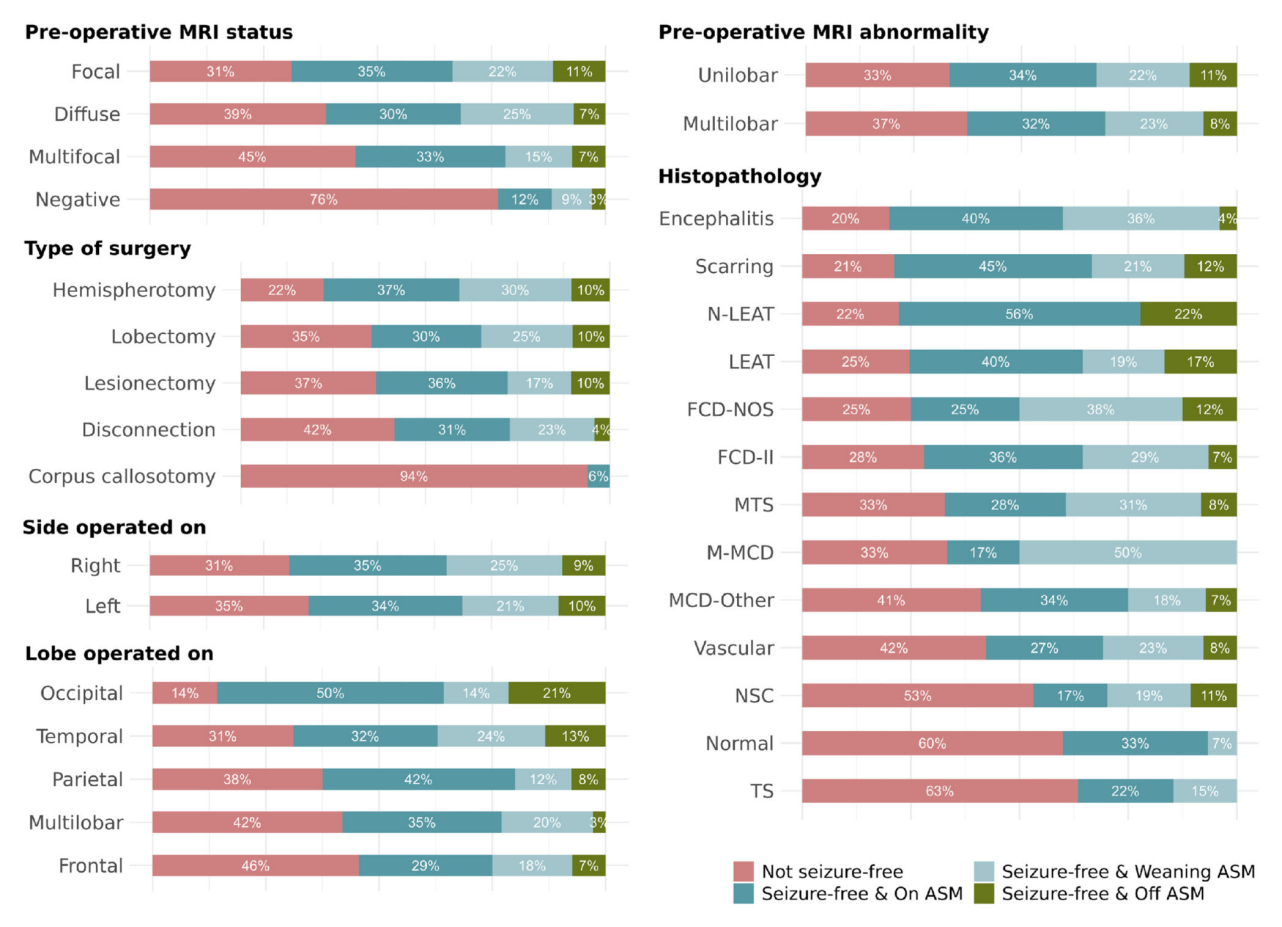
Breakdown of seizure freedom rates and post-operative antiseizure medication status by patient characteristics and surgery details. The figure provides an overview of seizure freedom rates and post-operative ASM status, which can used in clinical practice and in discussion with parents and patients. Abbreviations: ASM = Antiseizure medication; DNET = Dysembryoplastic neuroepithelial tumor; FCD-II = Focal cortical dysplasia type II; FCD-NOS = Focal cortical dysplasia not otherwise specified; LEAT = Low-grade epilepsy-associated tumor; MCD-Other = Malformation of cortical development-other; M-MCD = Mild malformation of cortical development; MTS = Mesial temporal sclerosis; N-LEAT = Non-low-grade epilepsy associated tumor; TS = Tuberous sclerosis.

Of the patients who were seizure-free at one-year follow-up, 51% were on ASM, 34% were weaning ASM, and 15% were off ASM. All seizure-free patients in the palliative procedures group were still on ASM. As in the case of seizure outcome, post-operative ASM status varied depending on patient characteristics (Figure 4). Children were also, over time, more likely to be weaning or off ASM at one-year follow-up (OR 1.04 [95% CI=1.01-1.08], p=0.013) (Figure 3C).

### Deaths

At time of final review (1 April 2023), 11 (1%) patients were deceased. None of these patients died during surgery. Eight of these patients had undergone a single surgery, whereas three had undergone a second surgery. Time from last surgery to death ranged from three days to 20.3 years (median=7.0, IQR=3.5-11.3 years). There was no evidence of surgical complication nor was there any evidence of cerebral swelling or herniation in the case of the patient who died three days after surgery, and with ongoing seizures it was concluded that their death was due to SUDEP (sudden unexpected death in epilepsy). All other deaths occurred more than 1.5 years after surgery. There was no information regarding the cause of these deaths, but all patients were experiencing seizures at one-year follow-up.

## Discussion

We studied the evolution of pediatric epilepsy surgery at our tertiary center from 2000 to 2018. To our knowledge, this represents one of the largest single-center study of pediatric epilepsy surgery to date, and the first attempt to examine trends in pediatric epilepsy surgery in the UK. We demonstrated an increase in the annual number of referrals and surgeries but found that children continue to be referred for pre-surgical evaluation after too many unsuccessful trials of antiseizure medications. We also demonstrated an improvement in seizure freedom rates, but only after an observed increase in complex cases was accounted for. Finally, we showed an increase in the likelihood of children weaning or being off ASM after surgery, and an expansion in genetic testing.

### Seizure freedom rates have improved over time but only after adjustment for an observed increase in complex cases

Excluding palliative procedures, we found that 67% of patients were seizure-free (including no auras) at one-year follow-up after surgery. This is in line with a recent systematic review investigating outcomes after pediatric epilepsy surgery, which reported seizure freedom rates of 51-76% for non-palliative procedures.^1^ Still, past studies have used variable definitions of seizure freedom, making it difficult to make a direct comparison. Critically, some studies have adopted definitions of seizure freedom that allow for the occurrence of non-disabling seizures. This can, in comparison to more stringent definitions, increase seizure freedom rates by as much as 20%.^9^

At first glance, we found that seizure freedom rates have remained stable over time. This observation was surprising, as it seemed to indicate that major advancements in pre-surgical assessment, as well as greater clinical experience, had not translated into comparable improvements in seizure freedom rates. At the same time, however, we also found an increase in the proportion of patient characteristics that could be considered complex: patients with diffuse and multifocal pre-operative MRI abnormalities, disconnective surgeries, and histopathology diagnoses of non-specific epilepsy-associated changes. Previous studies performed in pediatric epilepsy surgery patients have hypothesized that an increase in case complexity may have counteracted an improvement in seizure freedom rates over time.^8,14,17^ Indeed, when we adjusted for the observed increase in complex cases, we demonstrated that the likelihood of children achieving seizure freedom increased over time.

### Children are now more likely to be weaned off antiseizure medication after surgery compared to previously

Over time, we found an increase in the likelihood of children being weaned off ASM or on no ASM at one-year follow-up after surgery. This shift in ASM withdrawal policy may have been motivated by participation in a large multi-center study, which showed that early ASM withdrawal after surgery does not affect long-term seizure outcome^18^, as well as the known benefits of ASM cessation on cognition in children.^2^

Two studies have previously investigated temporal trends in ASM withdrawal after pediatric epilepsy surgery. Lamberink and colleagues^8^ - like us - found an increase in the proportion of patients weaned off ASM and no change in seizure freedom rates. Correspondingly, Hemb and colleagues^13^ reported a decrease in the proportion of patients weaned off ASM alongside an improvement in seizure freedom rates. The authors further concluded that their adoption of a more conservative ASM withdrawal policy may have contributed to their improvement in seizure freedom rates. Simultaneously, our shift toward an earlier ASM withdrawal policy may have served to unmask patients in whom surgery was not curative sooner.^18^

### Time elapsed between epilepsy onset and surgery has remained unchanged

Despite increases in referral and surgical volumes, we found no change in duration of epilepsy or the total number of different ASMs trialed from epilepsy onset to pre-surgical evaluation, with around 50% of the cohort having been trialed on ≥5 different ASMs, and nearly 25% trialed on ≥7 over the course of their epilepsy. This is surprising, considering the mounting evidence that shorter duration of epilepsy prior to surgery leads to better seizure outcome.^19^ It also exceeds national^20^ and international^21^ guidelines, which state that referral for surgical evaluation should be made as soon as drug-resistance, defined as a “failure of adequate trials of two tolerated and appropriately chosen ASM schedules (whether a monotherapies or in combination) to achieve seizure freedom”^22^, is ascertained. At the same time, it mirrors the findings of previous studies, which have similarly failed to show a reduction in epilepsy duration over time.^8,13^ One possible explanation could be referral hesitancy, driven either by the physician’s reluctance to refer patients for pre-surgical evaluation, or by the patient or family declining the recommendation for pre-surgical evaluation.^23–25^ There is thus a need to increase awareness in families, as well as local health professionals, on the safety and benefits of epilepsy surgery, so this is viewed as an early intervention rather than a last resort.^21,26^

### The potential role of genetic testing in pre-surgical planning

Genetic testing of epilepsy patients has expanded considerably in the past decades, primarily through the adoption of next generation sequencing techniques. As a result, an increasing number of epilepsy genes have been identified. We found a genetic cause of epilepsy in 34 patients. These patients showed significantly lower surgical success compared to those with a negative genetic test.

Researchers have proposed that genetic markers could serve as novel predictors of surgical success.^15^ However, with the exception of tuberous sclerosis complex^27^, little is known regarding the rate of surgical success in patients with a genetic diagnosis. Indeed, we report for the first time post-operative seizure outcomes for six genetic causes of epilepsy: *COL4A1*, *GRIN2B*, *NEXMIF*, *NSD1*, *SCN2A* and *SLC9A6*. None of these patients became seizure-free through surgery. We also add to existing, albeit scarce, literature by reporting outcomes for patients with *KRIT1*, *SCN1A* and *DEPDC5* pathogenic variants.^15,28–33^

In some patients, a genetic diagnosis is unlikely to affect surgery candidate selection. For example, diagnoses of tuberous sclerosis complex and multiple cavernoma (due to *KRIT1*), can often be made based on neuroimaging and clinical criteria; genetic testing is primarily performed to confirm diagnosis, inform surveillance for co-morbidities, and allow for family counselling. In other patients, such as those with a *SCN2A* pathogenic variant, a genetic diagnosis may indeed influence the decision to proceed with surgery, or at the very least affect estimates of seizure reduction by surgery. However, in our cohort, one third of patients with a genetic cause of epilepsy did not receive their diagnosis until after surgery.

### Limitations

The primary limitation of our study is that it is a single-center study, and thus influenced by local and national attitudes and practices. The generalizability of some of our findings may therefore be limited. However, in comparison to multi-center studies, we are able to present a cohort that is unique in its richness of data. As such, it can support other centers in their refinement of existing surgery candidate selection processes.

A second possible limitation is our definition of surgical success: we dichotomized patients as being either seizure-free or not seizure-free. We are, as a result, unable to discern whether patients who did not achieve seizure freedom still showed a significant improvement in seizure burden. This is especially problematic in patients who underwent palliative procedures of corpus callosotomy and multiple subpial transections, where the aim of surgery is a reduction in seizure burden and/or frequency rather than seizure freedom. Our definition of seizure outcome was, however, adopted to allow us to compare our findings with those of previous studies, and avoid ambiguous terminology such as “disabling” versus “nondisabling” seizures.

A related limitation is that we are only able to provide one-year post-operative follow-up. This is because only one-year follow-up is commissioned by the National Health Service (NHS; the publicly funded healthcare system in England), and therefore only one-year follow-up is performed for all epilepsy surgery patients at our center. Ideally, longer term follow-up, such as three- or five-year follow-up, should also be included when evaluating outcomes after epilepsy surgery.

Another limitation of our study is that we did not have data from the referring centers to determine the exact time-point at which medication-resistance was established. We have mitigated for this by counting the total number of different ASMs trialed from epilepsy onset to pre-surgical evaluation. Due to the lack of data from referral centers, we were also unable to determine the nature for why patients are not referred for epilepsy surgery sooner, which would help to identify, and ultimately overcome, potential barriers to early referral for epilepsy surgery.

Finally, it is important to note that only 15% of our cohort underwent genetic testing. Our findings related to the poor prognostic outlook for surgery patients with a genetic cause of epilepsy may, therefore, not be representative of the cohort as a whole, or indeed of epilepsy surgery patients in general. We are also unable to comment on whether a positive genetic finding may have resulted in a patient not being put forward for surgery. This is still one of the largest cohorts of epilepsy surgery patients to have undergone genetic testing to date. It therefore provides incentive for performing genetic testing on all epilepsy surgery patients, to establish the true prognostic value of genetic testing in pre-surgical evaluation.

## Conclusions

The number of children with epilepsy being treated with surgery has increased substantially over the past two decades. Although seizure freedom rates overall remain similar to those of 20 years ago, we show that they have improved when the increase in complex cases is accounted for. Nevertheless, we have identified several areas for potential improvement in surgical outcomes. Despite current guidelines urging prompt referral, referral for surgical evaluation comes late, as epilepsy duration remains protracted and the total number of different ASMs that patients have trialed since epilepsy onset continues to exceed guidelines. Furthermore, the advent of genetic testing may help to identify patients in whom surgery is far less likely to prove successful. There is therefore an urgent need to assess referral and diagnostic practices, to allow more children to be referred for early evaluation and, in extension, an earlier chance of seizure freedom.

## Supplementary Material

Supplementary Material

## Figures and Tables

**Table 1:** Demographic information, epilepsy characteristics, pre-operative MRI findings, genetic findings, surgery types, and histopathology diagnoses (N = 859).

**Sex** N (% of total sample)	
Females	402 (47)
Males	455 (53)
Missing data	2 (<1)
**Ethnicity** N (% of total sample, % according to 2011 Census for England and Wales)	
Asian	86 (10, 8)
Black	28 (3, 3)
Mixed	17 (2, 2)
Other	22 (3, 1)
White	595 (69, 86)
Ethnicity not asked or given	111 (13, NA)
**Epilepsy characteristics** years, median [IQR] (range)	
Age at first seizure ^a^	1.3 [0.3-4.0] (0-15.6)
Age of epilepsy onset	1.7 [0.4-4.9] (0-15.6)
Age at surgery	8.5 [4.6-13.4] (0.1-21.5)
Duration of epilepsy	5.1 [2.7-8.5] (0-20.7)
**Antiseizure medication (ASM)**	
**Number of ASMs that the patient was receiving at time of pre-surgical evaluation, mean [SD] (range)**	2.4 [1.0] (0-7)
0	10 (1)
1	145 (17)
2	335 (39)
3	251 (29)
4+	94 (11)
Missing data	24 (3)
**Total number of ASMs that the patient had trialed from time of epilepsy onset to time of pre-surgical evaluation, mean [SD] (range)**	5.0 [2.5] (0-17)
0	2 (<1)
1-2	108 (13)
3-4	286 (33)
5-6	226 (25)
7-8	129 (15)
9+	68 (8)
Missing data	40 (5)
**Pre-operative MRI findings** N (% of total sample)	
**Type of MRI abnormality**	
A. Focal	447 (52)
B. Non-focal	360 (42)
Diffuse	267 (31)
Multifocal	93 (11)
C. Negative	43 (5)
D. Missing data	9 (1)
**Extent of MRI abnormality**	
A. Unilobar	435 (51)
B. Multilobar	372 (43)
C. Negative	43 (5)
D. Missing data	9 (1)
**Genetic findings** N (% of total sample)	
Pathogenic/likely pathogenic SNV	22 (3)
CNV	12 (1)
Benign/likely benign SNV	29 (3)
No variant identified	62 (7)
No test administered	734 (85)
**Type of surgery** N (% of total sample)	
**Surgical disconnections**	309 (36)
A. Palliative procedures	68 (8)
Corpus callosotomy	63 (7)
Multiple subpial transections	5 (<1)
B. Non-palliative procedures	241 (28)
Hemi spherotomy	202 (24)
Disconnection	39 (5)
**Surgical resections**	520 (61)
A. Lesionectomy	309 (36)
B. Lobectomy	211 (25)
**Combined procedures**	24 (3)
A. Disconnection + Lobectomy	17 (2)
B. Lobectomy + Lesionectomy	6 (<1)
C. Disconnection + Lesionectomy	1 (<1)
Abandoned procedures	3 (<1)
Missing data	3 (<1)
**Side operated on** N (% of total sample)	
A. Left	414 (48)
B. Right	372 (43)
C. Abandoned procedures	3 (<1)
D. Missing data	3 (<1)
E. Not applicable ^b^	67 (8)
**Lobe operated on** N (% of total sample)	
A. Unilobar procedures	485 (56)
Temporal	273 (32)
Frontal	149 (17)
Parietal	46 (5)
Occipital	15 (2)
Insular	2 (<1)
B. Multilobar procedures	84 (10)
C. Abandoned procedures	3 (<1)
D. Missing data	3 (<1)
E. Not applicable ^c^	284 (33)
**Histopathology** N (% of total sample)	
LEAT	151 (18)
FCD-II	115 (13)
MTS	70 (8)
Scarring	67 (8)
MCD-Other	62 (7)
NSC	49 (6)
Tuberous sclerosis	46 (5)
Vascular	32 (4)
Rasmussen encephalitis	29 (3)
Normal result	19 (2)
N-LEAT	11 (1)
FCD-NOS	9 (1)
M-MCD	6 (1)
Histopathology not collected or report not available	193 (22)

a Age at first seizure and Age of epilepsy onset were kept distinct to account for early, isolated occurrences of febrile seizures (see Supplementary Material p. 2).

b Not applicable was assigned to corpus callosotomy procedures as well as focal resections that involved the removal of a hypothalamic hamartoma.

c Not applicable was assigned to hemispherotomy, corpus callosotomy and multiple subpial transection procedures, as well as focal resections that involved the removal of a hypothalamic hamartoma.

Abbreviations: ASM = Antiseizure medication; CNV = Copy number variation; DNET = Dysembryoplastic neuroepithelial tumor; FCD-II = Focal cortical dysplasia type II; FCD-NOS = Focal cortical dysplasia not otherwise specified; IQR = Interquartile range; LEAT = Low-grade epilepsy-associated tumor; MCD-Other = Malformation of cortical development-other; M-MCD = Mild malformation of cortical development; MTS = Mesial temporal sclerosis; NA = Not applicable; N-LEAT = Non-low-grade epilepsy-associated tumor; NSC = Non-specific epilepsy-associated changes; SNV = Single nucleotide variation.

**Table 2:** Pathogenic and likely pathogenic single nucleotide variants (SNVs) and their associated pre-operative MRI findings, surgery types, histopathology diagnoses, and post-operative seizure outcomes.

Variant	Gene function	Inheritance	Classification	ACMG criteria	MRI findings	Procedure	Histopathology	Time of test	Outcome
*COL4A1* c.3592G>A p.(Gly1198Arg)	Collagen subunit	*De novo*	Uncertain clinical significance – warm 3	PM2_Mod; PM6_sup; PP3	Periventricular leukomalacia	Corpus callosotomy	Not collected	Results known at pre-surgical evaluation	N-SF
*DEPDC5* c.280-10T>G	mTOR pathway regulator	Unknown	Uncertain clinical significance – warm 3	PM2_Mod; PP3	Focal cortical dysplasia	Lesionectomy	Not available	After surgery	N-SF ^a^
*GRIN2B* c.2453T>C; p.(Met818Thr)	Glutamate receptor component	*De novo*	Likely pathogenic	PM2_Mod; PP3; PP2; PM6_Mod; PS4_Sup	Polymicrogyria	Hemispherotomy	Polymicrogyria	After surgery	N-SF
*KRIT1* c.1878dupA, p.(Gln627Thrfs *28)	Microtubule associated protein associated with formation of cerebral cavernous malformations	*De novo*	Pathogenic	PVS1_Very strong; PM2_Mod; PM6_Sup	Cavernoma	Lesionectomy	Cavernoma	After surgery	NR ^b^
*KRIT1* c.2043del p.(Lys682Serfs* 25)	Microtubule associated protein associated with formation of cerebral cavernous malformations	Strong family history	Likely pathogenic	PVS1_Strong + PM2_Mod	Cavernoma	Lobectomy	Cavernoma	Results known at pre-surgical evaluation	SF ^b^
*NEXMIF* c.1433_1434del; p.(Tyr478Trpfs* 12)	Proposed role in neuronal morphogenesis, migration, and synapse formation	*De novo*	Pathogenic	PVS1_Very strong: PM2_Mod; PS2_Mod	Negative	Corpus callosotomy	Not collected	Results known at pre-surgical evaluation	N-SF
*NSD1* c.1237-6T>G het	Histone methyltransfer ase	Unknown	Uncertain clinical significance – warm 3	PM2_Mod; PP3	Undetermined lesion	Lobectomy	DNET	After surgery	N-SF
*SCN1A* c.4888G>A (p.Val1630Met)	Sodium channel component	*De novo*	Likely pathogenic	PM2_Mod; PM5_Mod; PM6_Sup; PP3; PP2	Hippocampal sclerosis	Lobectomy	Hippocampal sclerosis	Results known at pre-surgical evaluation	SF ^c^
*SCN1A* c.652T>C p.(Phe218Leu)	Sodium channel component	Maternally inherited (one sibling also has the variant; unaffected apart from single febrile seizure)	Likely pathogenic	PM2_Mod; PP3; PP2; PS4_Mod	Hippocampal sclerosis	Lobectomy	Hippocampal sclerosis	Results known at pre-surgical evaluation	N-SF ^c^
*SCN2A* c.4841T>C p.(Leu1614Pro)	Sodium channel component	*De novo*	Likely pathogenic	PM2_Mod; PP3; PP2; PS4_Sup; PM6_Sup	Polymicrogyria + Cortical dysplasia	Disconnection + Lobectomy	Polymicrogyria + Hippocampal sclerosis	After surgery	N-SF
*SLC9A6* c.1222_1226del p.(His408Asnfs *2)	Sodium/Hydro gen Ion exchange channel	*De novo*	Pathogenic	PVS1_Very strong; PM2_Mod; PM6_Sup	Undetermined abnormality	Corpus callosotomy	Not collected	After surgery	N-SF
*TSC1* c.2283C>G p.(Tyr761*)	mTOR pathway regulator	Paternally inherited (family history of TSC)	Pathogenic	PVS1_Very strong; PM2_Mod; PS4_Mod	Tuberous sclerosis	Lesionectomy	Tuberous sclerosis	Results known at pre-surgical evaluation	N-SF
*TSC1* c.2593C<T (p.Gln865*)	mTOR pathway regulator	Paternally inherited	Pathogenic	PVS1_Very strong; PM2_Mod	Tuberous sclerosis	Lesionectomy	Tuberous sclerosis	Results known at pre-surgical evaluation	SF
*TSC1^d^*	mTOR pathway regulator	Father has a history of epilepsy and clinical signs suggestive of TSC	Variant information missing	Variant information missing	Tuberous sclerosis	Lobectomy	Tuberous sclerosis	Results known at pre-surgical evaluation	N-SF
*TSC2* c.2251C>T (p.Arg751*)	mTOR pathway regulator	Unknown	Pathogenic	PVS1_Very strong: PM2_Mod; PS4_Mod	Tuberous sclerosis	Lobectomy	Tuberous sclerosis	Results known at pre-surgical evaluation	N-SF
*TSC2* c.2713C>T p.(Arg905Trp)	mTOR pathway regulator	*De novo*	Pathogenic	PM2_Mod; PS4_Mod; PM6_Strong; PM5_Mod; PP3	Tuberous sclerosis	Lesionectomy	Tuberous sclerosis	Results known at pre-surgical evaluation	SF
*TSC2* c.4006-1G>A	mTOR pathway regulator	Inherited from an affected father	Pathogenic	PVS1_Very strong: PM2_Mod	Focal cortical dysplasia	Lobectomy	Focal cortical dysplasia type II	Results known at pre-surgical evaluation	SF
*TSC2* c.4927A>C p.(Asn1643His)	mTOR pathway regulator	Unknown	Likely pathogenic	PM2_Mod; PS4_Mod; PM5_Mod; PP3	Tuberous sclerosis	Lesionectomy	Tuberous sclerosis	Results known at pre-surgical evaluation	N-SF
*TSC2* deletion of exon 32	mTOR pathway regulator	Unknown	Uncertain clinical significance	PVS1_Mod; PM2_Mod	Tuberous sclerosis	Lesionectomy	Tuberous sclerosis	Results known at pre-surgical evaluation	NR
*TSC2* ^d^	mTOR pathway regulator	*De novo*	Variant information missing	Variant information missing	Tuberous sclerosis + SEGA	Lesionectomy	SEGA	After surgery	N-SF
*TSC2* ^d^	mTOR pathway regulator	Variant information missing	Variant information missing	Variant information missing	Tuberous sclerosis + SEGA	Lesionectomy	Tuberous sclerosis	Results known at pre-surgical evaluation	N-SF
*TSC2* ^d^	mTOR pathway regulator	*De novo*	Variant information missing	Variant information missing	Tuberous sclerosis	Lesionectomy	Tuberous sclerosis	After surgery	N-SF

ACGM = The American College of Medical Genetics and Genomics; NR = Not reported; N-SF = Not seizure-free; SEGA = Subependymal giant cell astrocytoma; SF = Seizure-free; TSC = Tuberous sclerosis complex.

a 6/11 (55%) patients with a pathogenic variant of *DEPDC5* have previously been reported seizure-free after surgery.^15,30–32^

b 2/3 (67%) patients with a pathogenic variant of *KRIT1* have previously been reported seizure-free after surgery.^15^

c 4/16 (25%) patients with a pathogenic variant of *SCN1A* have previously been reported seizure-free after surgery.^28,29,33^

d Variant information not available.

## Data Availability

The study’s data dictionary and analytic code will be made publicly available on GitHub (https://github.com/MariaEriksson/GOSH-2000-2018). The full data are not publicly available due to their sensitive nature. Deidentified data will be made available from the corresponding author upon reasonable request.

## Significance

Children with drug-resistant epilepsy continue to be put forward for surgery late, despite national and international guidelines urging prompt referral. Seizure freedom rates have improved over the past decades, but only after adjustment for a concurrent increase in complex cases. Finally, genetic testing in epilepsy surgery patients has expanded considerably over time and shows promise in identifying patients in whom surgery is unlikely to be successful.

## Key Points

- The number of children with epilepsy being referred for and treated with surgery has increased over the past two decades.
- Still, children continue to be put forward for surgery late and after too many unsuccessful trials of antiseizure medications.
- The annual proportion of cases that could be considered complex has increased over time.
- Current seizure freedom rates are similar to those of 20 years ago but show improvement when the increase in complex cases is accounted for.
- Future research should systematically evaluate the predictive value of genetic testing in pre-surgical evaluation.

## Glossary

ASM: antiseizure medication
CESS: Children’s Epilepsy Surgery Service
CI: confidence interval
CNV: copy number variation
IQR: interquartile range
OR: odds ratio
SNV: single nucleotide variation
SUDEP: sudden unexpected death in epilepsy
