# Supplementary material for: Pediatric epilepsy surgery from 2000 to 2018: Changes in referral and surgical volumes, patient characteristics, genetic testing, and post-surgical outcomes
Source: Epilepsia. Author manuscript; Available in PMC 2024 Apr 25. (PMC7615891; doi:10.1111/epi.17670)
Supplement: Supplementary Material [file EMS195582-supplement-Supplementary_Material.docx]

**Supplementary document accompanying the manuscript “Pediatric epilepsy surgery from 2000 to 2018:** **Changes in referral and surgical volumes, patient characteristics, genetic testing, and post-surgical outcomes”.**

Table of Contents

[Data set description and data classification 2](#_Toc138467527)

[1. Demographic variables 2](#_Toc138467528)

[1.1. Ethnicity 2](#_Toc138467529)

[2. Epilepsy characteristics 2](#_Toc138467530)

[2.1. Age at first seizure 2](#_Toc138467531)

[2.2. Age of epilepsy onset 2](#_Toc138467532)

[2.3. Age at surgery 2](#_Toc138467533)

[2.4. Duration of epilepsy 2](#_Toc138467534)

[2.5. Number of antiseizure medications at time of pre-surgical evaluation 2](#_Toc138467535)

[2.6. Total number of different antiseizure medications trialed from time of epilepsy onset to time of pre-surgical evaluation 3](#_Toc138467536)

[3. Pre-operative MRI findings 3](#_Toc138467537)

[3.1. Type of pre-operative MRI abnormality 3](#_Toc138467538)

[3.2. Extent of pre-operative MRI abnormality 3](#_Toc138467539)

[4. Genetic diagnoses 3](#_Toc138467540)

[5. Surgery details 4](#_Toc138467541)

[5.1. Type of surgery 4](#_Toc138467542)

[5.2. Side operated on 4](#_Toc138467543)

[5.3. Lobe operated on 4](#_Toc138467544)

[6. Histopathology diagnoses 4](#_Toc138467545)

[7. Seizure outcome 5](#_Toc138467546)

[8. Post-operative antiseizure medication status 5](#_Toc138467547)

[Patient flowchart 6](#_Toc138467548)

[National referral practices 7](#_Toc138467549)

[Temporal trends 8](#_Toc138467550)

[Changes in epilepsy characteristics over time 10](#_Toc138467551)

[Histopathology findings of MRI negative and previously MRI negative cases 11](#_Toc138467552)

[Genetic testing 12](#_Toc138467553)

[Seizure freedom rates 15](#_Toc138467554)

# Data set description and data classification

## Demographic variables

### Ethnicity

Patient ethnicity was coded as one of six possible groups: Asian, Black, Mixed, Other, White, or Ethnicity not asked or given. These groups were chosen in accordance with the 2011 Census for England and Wales.

## Epilepsy characteristics

### Age at first seizure

Age at first seizure was a continuous variable used to capture the age at which the child experienced their first seizure. This could have been either a febrile or afebrile seizure.

### Age of epilepsy onset

Age of epilepsy onset was a continuous variable used to capture the age at which the child was diagnosed with epilepsy. The variables “Age at first seizure” and ”Age of epilepsy onset” were kept distinct to account for early, isolated occurrences of febrile seizure(s), which would not have resulted in a diagnosis of epilepsy. For example, if a child experienced a single febrile seizure at 7 months and was subsequently seizure-free until the age of 5 years, at which time they experienced multiple afebrile seizures, the child’s age at first seizure would have been classified as 7 months, while their age of epilepsy onset would have been classified as 5 years. However, if the child had experienced afebrile seizures immediately following their febrile seizure at 7 months, their age at first seizure and age of epilepsy onset would have been the same.

### Age at surgery

Age at surgery was a continuous variable used to capture the age at which the child underwent their initial resective or disconnective surgery for epilepsy.

### Duration of epilepsy

Duration of epilepsy was a continuous variable used to capture the time elapsed between the child being diagnosed with epilepsy and undergoing surgery (“Age at surgery” - “Age of epilepsy onset” = “Duration of epilepsy”).

### Number of antiseizure medications at time of pre-surgical evaluation

We counted to the number of antiseizure medications (ASMs) that the patient was receiving at time of their pre-surgical evaluation. We did not include rescue medication (e.g. Midazolam) or medication for status epilepticus (e.g. Thiopentone) in this count.

### Total number of different antiseizure medications trialed from time of epilepsy onset to time of pre-surgical evaluation

We counted to the total number of different antiseizure medications (ASMs) that the patient had trialed from the time of their epilepsy onset to the time of their pre-surgical evaluation. We did not include rescue medication (e.g. Midazolam) or medication for status epilepticus (e.g. Thiopentone) in this count.

## Pre-operative MRI findings

### Type of pre-operative MRI abnormality

The patient’s pre-operative MRI scan was classified as either abnormal (MRI positive) or normal (MRI negative). The patient’s MRI scan was considered abnormal if any type of abnormality could be seen. The only instances when patients were considered ‘MRI negative’ were when no abnormality at all could be seen on their scan.

It was also noted if patients had previously been MRI negative but MRI positive at the time of their surgery. For example, patients may have been MRI negative on referral but not following imaging at GOSH, or an abnormality may have been identified on their scan during pre-surgical evaluation. MRI scans classified as abnormal were further classified as comprising a focal (e.g. focal cortical dysplasia type II), multifocal (e.g. tuberous sclerosis) or diffuse (e.g. Rasmussen encephalitis) abnormality.

### Extent of pre-operative MRI abnormality

The patient’s pre-operative MRI scan was also classified as having an abnormality that affected either one lobe (unilobar) or several lobes (multilobar).

## Genetic diagnoses

All genetic tests performed in children who underwent surgery were retrospectively retrieved. The reported genetic variants were reviewed by APC, an experienced clinical scientist with a specialization in molecular genetics. Variant classification was carried out using Alamut Visual version 2.15 (SOPHiA GENETICS, Lausanne, Switzerland). Population data (<https://gnomad.broadinstitute.org/>), in silico tools predictions, functional analysis, segregation studies, and previous reports were taken into account. Variants are classified using the American College of Medical Genetics (ACMG)/Association for Molecular Pathology (AMP) guidelines and Association for Clinical Genomic Science (ACGS) Best Practice Guidelines for Variant Classification in Rare Disease 2020 (<https://www.acgs.uk.com/quality/best-practice-guidelines/>).

Findings were discussed with LM, a clinical geneticist, and AM, a consultant pediatric neurologist. Variants were classified into five categories as per standard ACMG guidelines, namely: 1) benign, 2) likely benign, 3) uncertain significance, 4) likely pathogenic, and 5) pathogenic. We considered patients with variants classified as class 4 and 5, as well as class 3 “warm”, to have a genetic cause of epilepsy, in accordance with ACGS Best Practice Guidelines for Variant Classification in Rare Disease 2020 (<https://www.acgs.uk.com/quality/best-practice-guidelines/>).

## Surgery details

Surgeries were classified by visual inspection of pre- and post-operative MRI scans as well as with medical records describing the pre-operative intent, surgical procedure, and post-operative discharge. Surgeries were characterized according to type of surgery, side operated on, and lobe operated on.

### Type of surgery

Type of procedure performed: Lesionectomy, Lobectomy, Disconnection, Hemispherotomy, Corpus callosotomy, or Multiple subpial transections. Procedures comprising more than one type of procedure, so-called “Combined procedures”, were reported in tables as such, but re-classified for analysis purposes. For re-classification, the more extensive procedure was used. For example, a surgery comprising both a lobectomy and lesionectomy would have been re-classified as a lobectomy.

### Side operated on

Side operated on: Right or Left. When classifying “Side operated on”, “Not applicable” was assigned to corpus callosotomy procedures as well as focal resections that involved the removal of a hypothalamic hamartoma.

### Lobe operated on

Lobe operated on: Temporal, Frontal, Parietal, Occipital, Insular or Multilobar. When classifying “Lobe operated on”, “Not applicable” was assigned to hemispherotomy, corpus callosotomy and multiple subpial transections, as well as focal resections that involved the removal of a hypothalamic hamartoma.

## Histopathology diagnoses

Histopathology diagnosis was grouped into one of thirteen categories.

| **Histopathology diagnoses.** |
| --- |
| (1) FCD-II (focal cortical dysplasia type II) |
| (2) FCD-NOS (focal cortical dysplasia not otherwise specified) |
| (3) LEAT (low-grade epilepsy-associated tumor, including ganglioglioma, dysembryoplastic neuroepithelial tumor, pilocytic astrocytoma, and papillary glioneuronal tumor) |
| (4) M-MCD (mild malformation of cortical development, including focal cortical dysplasia type I) |
| (5) MCD-Other (including hypothalamic hamartoma, hemimegalencephaly, polymicrogyria, and focal cortical dysplasia type III) |
| (6) MTS (mesial temporal sclerosis, including hippocampal sclerosis, neuronal loss in the hippocampus/temporal lobe, gliosis in hippocampus/temporal lobe, granule cell dispersion, and Chaslin’s gliosis) |
| (7) N-LEAT (non-low-grade epilepsy-associated tumor, including astrocytoma, neurocytoma, and pleomorphic xanthoastrocytoma) |
| (8) Normal result |
| (9) NSC (non-specific epilepsy-associated changes) |
| (10) Rasmussen encephalitis |
| (11) Scarring (as result of an acute vascular event, i.e. hemorrhagic infarction, trauma, or infection in the past), |
| (12) TS (tuberous sclerosis) |
| (13) Vascular diagnoses (including cavernoma, meningioangiomatosis, as seen in Sturge-Weber syndrome, and arterio-venous malformations) |

## Seizure outcome

We classified patients as either seizure-free (including no auras) or not seizure-free at one-year follow-up. While seizure outcome following epilepsy surgery can be reported in different ways, we chose to characterize patients as either seizure-free or not seizure-free as this metric has been used by the majority of outcome studies published to date, and we wanted to ensure comparability with previous studies. We, furthermore, chose one-year follow-up, as opposed to last follow-up, because it is standard practice for GOSH to obtain seizure outcome for all epilepsy surgery patients at this time-point. One-year follow-up thus offers consistent data for all patients and avoids comparing patients who are at different stages of their post-operative trajectory, which can be problematic as seizure freedom rates are known to decline over time.

## Post-operative antiseizure medication status

We recorded if patients who were seizure-free at one-year follow-up were on, weaning or off antiseizure medication.

# Patient flowchart


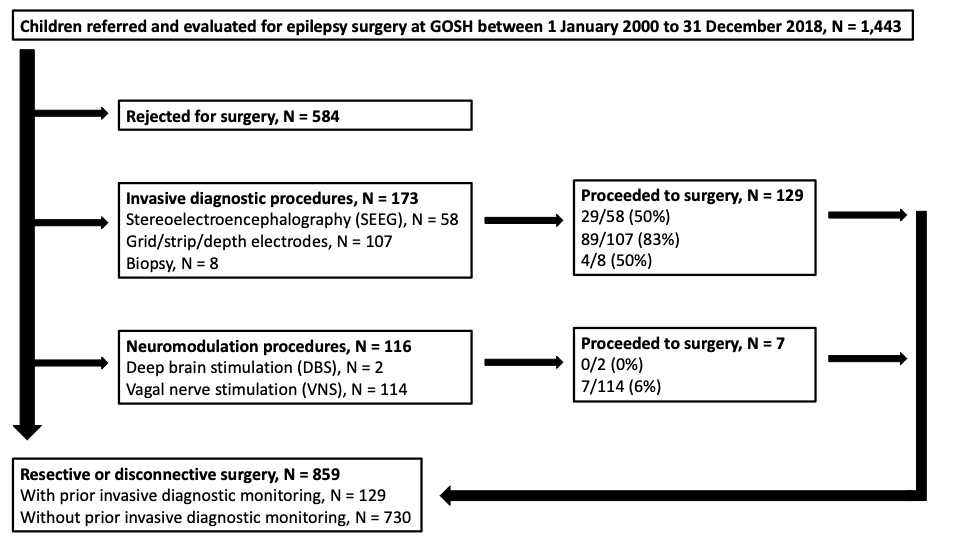


**Supplementary Figure 1. Flowchart of patient inclusion.**

In total, 1,443 children were referred and evaluated for epilepsy surgery at GOSH between 1 January 2000 and 31 December 2018. Of these, 859 went on to have surgical resection or disconnection and were included in the study. We excluded children who underwent neuromodulation procedures, as deep brain stimulation and responsive neurostimulation are not approved nor commissioned procedures for children with epilepsy in the UK. Two children did undergo deep brain stimulation, but these procedures were performed under exceptional and compassionate circumstances. We also excluded children who underwent thermocoagulation, as this is primarily used as a prognostic test rather than definitive treatment in the UK. If patients had undergone multiple resective and/or disconnective surgeries over the course of the study period, we included only their first surgery.

# National referral practices


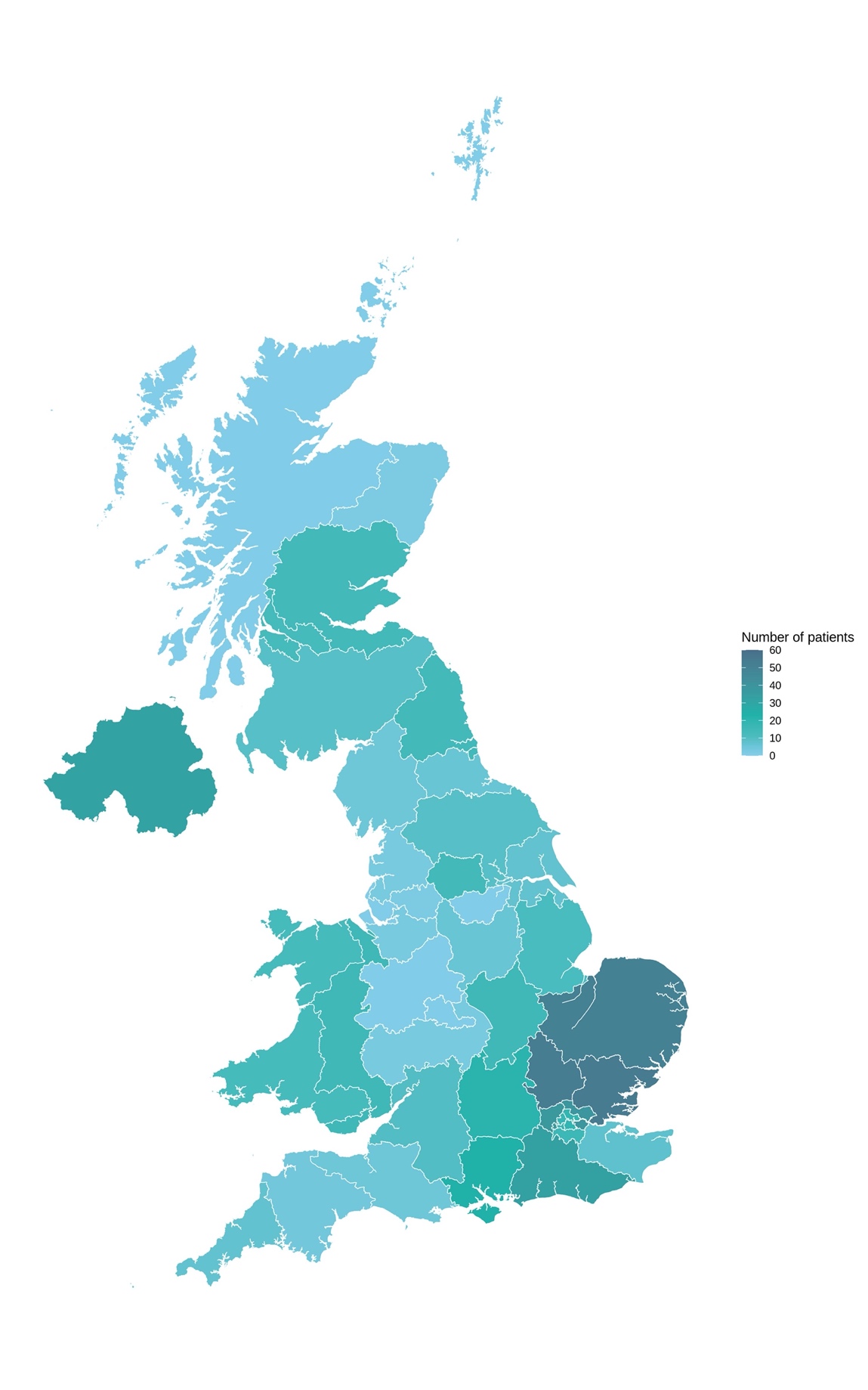


**Supplementary Figure 2.** Map of the UK showing where all surgical patients (2000-2018) had been referred from.

# Temporal trends

| **Supplementary Table 1. Temporal trends in referral and surgical volumes, pre-operative MRI findings, genetic findings, surgery types, and histopathology diagnoses.** We performed univariable negative binomial regression analyses, with correction for multiple comparison using Holm method. | | | | |
| --- | --- | --- | --- | --- |
|  | **Annual Change in Count %** (**95% CI**) | ***p*-Value *^a^*** | **Annual Change in Proportion %** (**95% CI**) | ***p*-Value *^a^*** |
| Referrals | 6.9 (5.3, 8.6) | <0.001 | - | - |
| Surgeries | 4.2 (2.9, 5.6) | <0.001 | - | - |
| **Pre-operative MRI findings** |  |  |  |  |
| **Type of MRI abnormality** |  |  |  |  |
| Focal | 2.1 (0.4, 3.8) | 0.036 | -2.2 (-3.9, -0.4) | 0.028 |
| Negative | 6.0 (0.3, 12.3) | 0.041 | 1.8 (-3.8, 8.1) | 0.537 |
| Non-focal | 7.2 (5.0, 9.5) | <0.001 | 2.9 (1.0, 5.0) | 0.015 |
| **Extent of MRI abnormality** |  |  |  |  |
| Unilobar | 3.0 (1.2, 4.8) | <0.001 | -1.3 (-3.0, 1.0) | 0.230 |
| Multilobar | 6.0 (3.6, 8.4) | <0.001 | 1.6 (-0.4, 3.6) | 0.230 |
| **Genetic findings** |  |  |  |  |
| Pathogenic SNV/CNV | 21.0 (12.2, 32.0) | <0.001 | 17.3 (8.4, 28.4) | <0.001 |
| Test performed | 23.5 (18.1, 30.0) | <0.001 | 19.9 (14.6, 25.9) | <0.001 |
| **Type of surgery** |  |  |  |  |
| Lobectomy | 2.5 (-0.1, 5.2) | 0.104 | -1.7 (-4.3, 1.0) | 0.965 |
| Lesionectomy | 4.2 (1.8, 6.6) | 0.002 | 0.1 (-2.2, 2.1) | 1.000 |
| Corpus callosotomy | 4.9 (0.0, 10.3) | 0.104 | 1.0 (-4.0, 5.6) | 1.000 |
| Hemispherotomy | 4.9 (1.9, 8.1) | 0.003 | 1.0 (-2.0, 3.3) | 1.000 |
| Disconnection | 14.0 (6.2, 23.0) | <0.001 | 9.3 (2.3, 17.5) | 0.040 |
| **Side operated on** |  |  |  |  |
| Right | 4.1 (2.1, 6.1) | <0.001 | -0.1 (-2.0, 1.8) | 1.000 |
| Left | 4.3 (2.8, 6.2) | <0.001 | 0.1 (-1.7, 2.0) | 1.000 |
| **Lobe operated on** |  |  |  |  |
| Temporal | 2.1 (-0.3, 4.6) | 0.243 | -2.1 (-4.4, 0.0) | 0.380 |
| Parietal | 3.6 (-1.7, 9.4) | 0.378 | -0.6 (-5.8, 5.1) | 1.000 |
| Occipital | 5.6 (-5.0, 18.2) | 0.378 | 1.1 (-8.9, 13.0) | 1.000 |
| Frontal | 5.9 (2.2, 9.7) | 0.008 | 1.7 (-1.7, 5.3) | 0.996 |
| Multilobar | 7.9 (3.7, 12.6) | 0.001 | 3.8 (-0.5, 8.3) | 0.380 |
| **Histopathology** |  |  |  |  |
| FCD-NOS | -23.7 (-39.4, -9.9) | 0.061 | -27.5 (-42.8, -14.0) | 0.012 |
| N-LEAT | -8.2 (-18.5, 2.6) | 0.876 | -12.3 (-22.5, -1.9) | 0.286 |
| Vascular | -1.2 (-7.3, 5.3) | 1.000 | -5.4 (-11.5, 1.1) | 0.900 |
| MCD-Other | 0.6 (-4.2, 5.7) | 1.000 | -3.6 (-8.0, 1.0) | 0.900 |
| MTS | 2.1 (-2.9, 7.5) | 1.000 | -1.9 (-7.1, 3.7) | 1.000 |
| LEAT | 2.7 (-0.2, 5.8) | 0.582 | -1.5 (-4.4, 1.5) | 1.000 |
| Normal result | 5.1 (-3.2, 14.7) | 1.000 | 0.9 (-7.3, 10.3) | 1.000 |
| Rasmussen encephalitis | 6.0 (-1.2, 14.0) | 0.770 | 1.6 (-5.8, 10.0) | 1.000 |
| Scarring | 6.3 (1.4, 11.7) | 0.117 | 2.1 (-2.5, 7.1) | 1.000 |
| FCD-II | 7.1 (3.1, 11.2) | 0.005 | 3.0 (-0.6, 6.8) | 0.900 |
| M-MCD | 9.8 (-5.5, 30.9) | 1.000 | 5.7 (-9.4, 26.7) | 1.000 |
| Tuberous sclerosis | 11.1 (5.0, 17.9) | 0.005 | 7.0 (1.0, 13.8) | 0.286 |
| NSC | 23.0 (11.6, 37.7) | <0.001 | 18.2 (7.7, 31.6) | 0.005 |
| Abbreviations: CI = Confidence interval; FCD-II = Focal cortical dysplasia type II; FCD-NOS = Focal cortical dysplasia not otherwise specified; LEAT = Low-grade epilepsy-associated tumor; MCD-Other = Malformation of cortical development other; M-MCD = Mild malformation of cortical development; MTS = Mesial temporal sclerosis; N-LEAT = Non-low-grade epilepsy-associated tumor; NSC = Non-specific epilepsy-associated changes.  *^a^* Corrected p-value. | | | | |

# Changes in epilepsy characteristics over time

| **Supplementary Table 2. Relationship between date of surgery and epilepsy characteristics.** Spearman’s rank-order correlation analyses were used to investigate the relationship between date of surgery, transformed into a numerical variable, and epilepsy characteristics of age of epilepsy onset, age at surgery, and duration of epilepsy at time of surgery. Correction for multiple comparison was performed using Holm method. | | |
| --- | --- | --- |
|  | **Spearman’s rank-order correlation** | ***p-*Value *^a^*** |
| Age of epilepsy onset | -0.03 | 1.00 |
| Age at surgery *^b^* | 0.01 | 1.00 |
| Duration of epilepsy *^b^* | 0.03 | 1.00 |
| *^a^* Corrected p-value.  *^b^* Controlling for age of epilepsy onset. | | |

# Histopathology findings of MRI negative and previously MRI negative cases

| **Supplementary Table 3. Histopathology findings of patients reported as “MRI negative” and “Previously MRI negative” at time of surgery (N=87; 10% of the entire cohort).** | |
| --- | --- |
| Histopathology N (% of sub-sample) | |
| **MRI negative at time of surgery (N=43)** | |
| NSC | 6 (14) |
| Normal | 2 (5) |
| FCD-II | 2 (5) |
| MTS | 1 (2) |
| No report *^a^* | 32 (74) |
| **MRI positive at time of surgery, but previously reported as MRI negative (N=44)** | |
| FCD-II | 14 (32) |
| NSC | 7 (16) |
| MTS | 5 (11) |
| LEAT | 4 (9) |
| FCD-NOS | 4 (9) |
| N-LEAT | 1 (2) |
| Vascular | 1 (2) |
| Normal | 1 (2) |
| No report | 7 (16) |
| *^a^* The high proportion of “No report” instances was due to the high proportion of MRI negative cases undergoing corpus callosotomy (28 of 43 MRI negative cases). | |
| Abbreviations: FCD-II = Focal cortical dysplasia type II; FCD-NOS = Focal cortical dysplasia not otherwise specified; LEAT = Low-grade epilepsy-associated tumor; MTS = Mesial temporal sclerosis; N-LEAT = Non-low-grade epilepsy-associated tumor; NSC = Non-specific epilepsy-associated changes. | |

# Genetic testing


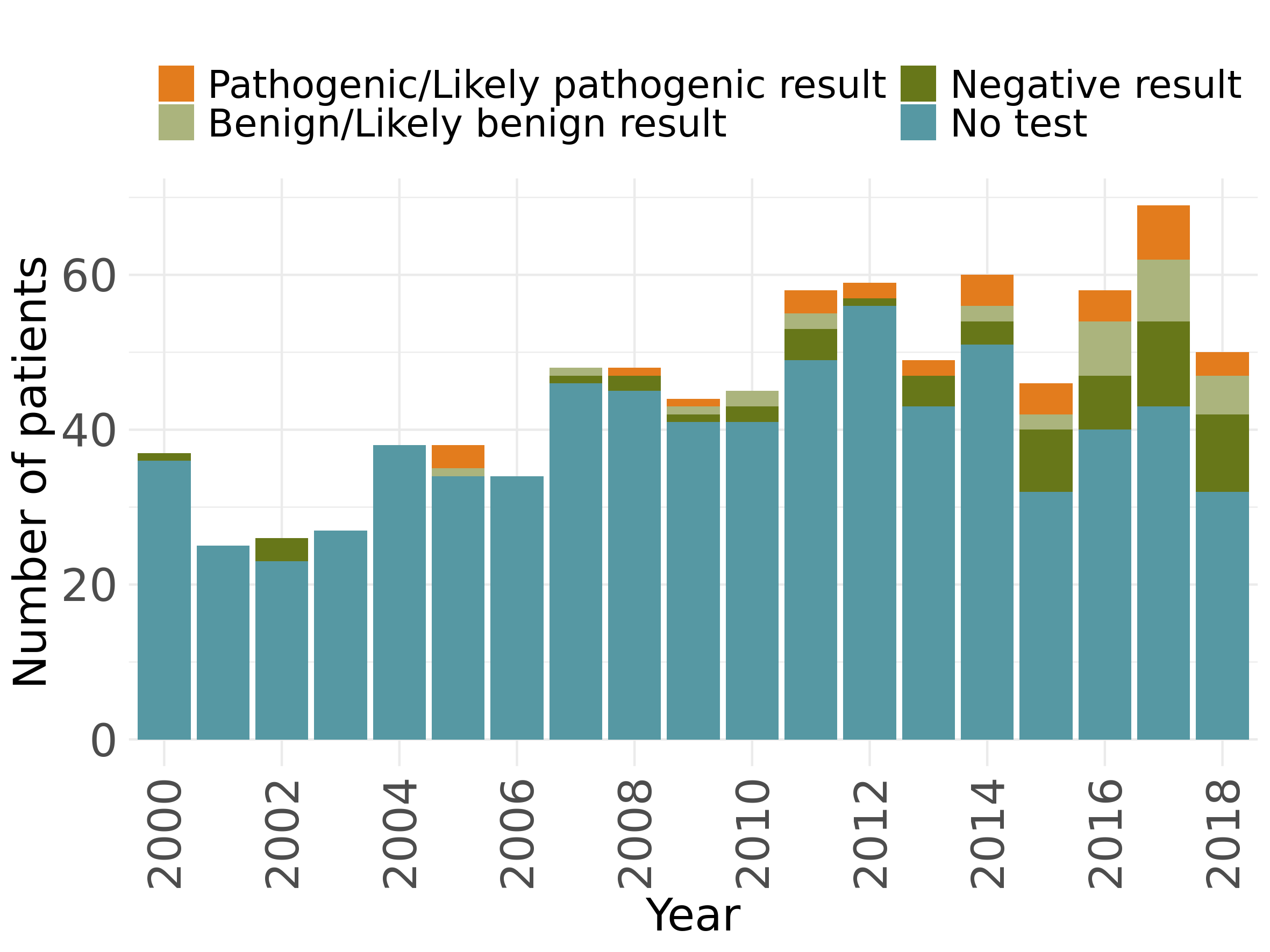


**Supplementary Figure 3. Temporal trends in genetic testing.** Changes in the number of patients who (1) underwent genetic testing, (2) had a negative result (no variant identified), (3) had a benign or likely benign variant, and (4) had a pathogenic or likely pathogenic variant.

| **Supplementary Table 4. Pathogenic and likely pathogenic copy number variations (CNVs) and their associated pre-operative MRI findings, surgery types, histopathology diagnoses, and post-operative seizure outcomes.** | | | | | | | |
| --- | --- | --- | --- | --- | --- | --- | --- |
| **Variant** | **Inheritance** | **Classification** | **MRI findings** | **Procedure** | **Histopathology** | **Time of test** | **Outcome** |
| Ring chromosome 22 | Presumed not inherited as parents healthy | Specific details of abnormality not available. May be contributing to phenotype. | Undetermined abnormality | Abandoned | Not collected | Results known at pre-surgical evaluation | NA |
| Chromosome 17 copy number loss 17q22-17q22.3 | *De novo* | May be contributing to phenotype. | Negative | Corpus callosotomy | Not collected | Results known at pre-surgical evaluation | N-SF |
| Chromosome 15  microdeletion 15q13.2-15q13.3 | Maternally inherited | Array finding consistent with phenotype. | Cortical dysplasia | Disconnection + Lesionectomy | Non-specific epilepsy-associated changes | Results known at pre-surgical evaluation | N-SF |
| Chromosome 2 copy number loss 2q14.3 | Paternally inherited | May be contributing to phenotype. | Infarct | Hemispherotomy | Hippocampal sclerosis | Results known at pre-surgical evaluation | SF |
| Chromosome 5 copy number loss involving *MCC2* gene | Maternally inherited | Specific details of abnormality not available. May be contributing to phenotype. | Focal cortical dysplasia | Lesionectomy | Focal cortical dysplasia type II | Results known at pre-surgical evaluation | N-SF |
| Chromosome 15 copy number gain 15q11.2-15q13.4 | *De novo* | Array finding consistent with phenotype. | Negative | Corpus callosotomy | Not collected | Results known at pre-surgical evaluation | NR |
| Chromosome 12 copy number gain | Maternally inherited | Specific details of abnormality not available. Contribution to phenotype unclear. | Cortical malformation | Lesionectomy | Polymicrogyria | After surgery | NR |
| Chromosome 1 copy number gain 1q22-1q22.3 | Inheritance unknown | Specific details of abnormality not available. Contribution to phenotype unclear. | Focal cortical dysplasia | Lesionectomy | Focal cortical dysplasia type II | Results known at pre-surgical evaluation | N-SF |
| Mosaic trisomy 13 | Not inherited as mosaic | Specific details of abnormality not available. May be contributing to phenotype. | Focal cortical dysplasia | Lobectomy | Hippocampal sclerosis | Results known at pre-surgical evaluation | SF |
| Chromosome 1 copy number loss 1p36 | *De novo* | Array finding consistent with phenotype. | Undetermined abnormality | Corpus callosotomy | Not collected | Results known at pre-surgical evaluation | N-SF |
| Chromosome 7 copy number gain 7q11.23 including *ELN* and *LIMK1* genes | Not present in mother, paternal status unknown | Contribution to phenotype unclear. | Hemimegalencephaly | Hemispherotomy | Hemimegalencephaly | After surgery | N-SF |
| Chromosome 2 copy number loss  2p24.3 including *MYCN* gene | Unknown | Contribution to phenotype unclear. | Infarct | Hemispherotomy | Not available | After surgery | SF |
| Abbreviations: NR = Not reported; N-SF = Not seizure-free; SF = Seizure-free. | | | | | | | |

# Seizure freedom rates

| **Supplementary Table 5. Seizure freedom rates by patient demographics, pre-operative MRI findings, genetic findings, surgery types, and histopathology diagnoses.** | |
| --- | --- |
|  | **Patients seizure-free %** (**95% CI**) |
| **Sex** |  |
| Females | 64.3 (59.2, 69.3) |
| Males | 61.8 (57.0, 66.5) |
| **Pre-operative MRI findings** |  |
| **Type of MRI abnormality** |  |
| Focal | 68.9 (64.4, 73.4) |
| Diffuse | 61.3 (55.0, 67.6) |
| Multifocal | 54.9 (43.9, 65.9) |
| Negative | 23.5 (8.5, 38.6) |
| **Extent of MRI abnormality** |  |
| Unilobar | 66.8 (62.1, 71.4) |
| Multilobar | 62.5 (56.8, 67.5) |
| **Genetic findings** |  |
| Pathogenic/likely pathogenic SNV | 25.0 (4.2, 45.8) |
| Benign/likely benign SNV | 30.0 (12.6, 47.4) |
| CNV | 33.3 (0.0-71.8) |
| No variant identified | 50.9 (37.3, 64.5) |
| No test administered | 67.0 (63.3, 70.6) |
| **Type of surgery** |  |
| Hemispherotomy | 77.6 (71.3, 83.8) |
| Lobectomy | 64.6 (57.9, 71.4) |
| Lesionectomy | 63.3 (57.6, 69.0) |
| Disconnection | 58.3 (43.9, 72.8) |
| Corpus callosotomy | 6.0 (0.0, 12.8) |
| **Side operated on** |  |
| Right | 69.4 (64.4, 74.4) |
| Left | 65.1 (60.3, 70.0) |
| **Lobe operated on** |  |
| Occipital | 85.7 (64.7, 100.0) |
| Temporal | 69.0 (63.2, 74.8) |
| Parietal | 62.4 (46.8, 78.2) |
| Multilobar | 58.1 (46.6, 69.6) |
| Frontal | 54.4 (45.9, 62.9) |
| **Histopathology** |  |
| Rasmussen encephalitis | 80.0 (63.1, 96.9) |
| Scarring | 78.8 (68.7, 88.9) |
| N-LEAT | 77.8 (43.9, 100.0) |
| LEAT | 75.4 (68.1, 82.6) |
| FCD-NOS | 75.0 (36.3, 100.0) |
| FCD-II | 72.0 (63.3, 80.6) |
| MTS | 67.2 (55.1, 79.3) |
| M-MCD | 66.7 (12.5, 100.0) |
| MCD-Other | 58.9 (45.6, 72.2) |
| Vascular | 57.7 (37.3, 78.0) |
| NSC | 46.8 (32.0, 61.6) |
| Normal result | 40.0 (11.9, 68.1) |
| Tuberous sclerosis | 36.6 (21.2, 52.0) |
| Seizure outcome at one-year post-operative follow-up was available for 751 patients: 3 (<1%) patients had a surgery that was abandoned, 2 (<1%) patients underwent a second surgery within the first year of their initial surgery, 34 (4%) patients were missing follow-up, and 69 (8%) patients had been followed up outside the specified time frame.  Abbreviations: CI = Confidence interval; FCD-II = Focal cortical dysplasia type II; FCD-NOS = Focal cortical dysplasia not otherwise specified; LEAT = Low-grade epilepsy-associated tumor; MCD-Other = Malformation of cortical development-other; M-MCD = Mild malformation of cortical development; MTS = Mesial temporal sclerosis; N-LEAT = Non-low-grade epilepsy-associated tumor; NSC = Non-specific epilepsy-associated changes. | |

| **Supplementary Table 6. Probability of achieving seizure freedom over time, by histopathology diagnosis.** Multivariable logistic regression with seizure freedom at one-year follow-up as outcome. Diagnoses of focal cortical dysplasia not otherwise specified and mild malformation of cortical development were excluded from analysis due to small N. | | | |
| --- | --- | --- | --- |
| **Variable** | **OR** | **95% CI** | ***p*-Value** |
| Interaction Encephalitis * Procedure date | (ref) |  |  |
| Interaction TS * Procedure date | 0.90 | (0.67, 1.14) | 0.400 |
| Interaction Vascular * Procedure date | 0.89 | (0.66, 1.13) | 0.365 |
| Interaction NSC * Procedure date | 0.87 | (0.64, 1.14) | 0.318 |
| Interaction LEAT * Procedure date | 0.85 | (0.64, 1.04) | 0.150 |
| Interaction Normal * Procedure date | 0.85 | (0.62, 1.12) | 0.265 |
| Interaction MTS * Procedure date | 0.81 | (0.62, 1.01) | 0.092 |
| Interaction FCD-II * Procedure date | 0.81 | (0.62, 1.00) | 0.075 |
| Interaction Scarring * Procedure date | 0.81 | (0.61, 1.01) | 0.088 |
| Interaction N-LEAT * Procedure date | 0.79 | (0.50, 1.21) | 0.262 |
| Interaction MCD-Other * Procedure date | 0.78 | (0.59, 0.97) | 0.040 |
| Abbreviations: CI = Confidence interval; FCD-II = Focal cortical dysplasia type II; LEAT = Low-grade epilepsy-associated tumor; MCD-Other = Malformation of cortical development other; MTS = Mesial temporal sclerosis; N-LEAT = Non-low-grade epilepsy-associated tumor; NSC = Non-specific epilepsy-associated changes; OR = Odds ratio. TS = Tuberous sclerosis. | | | |


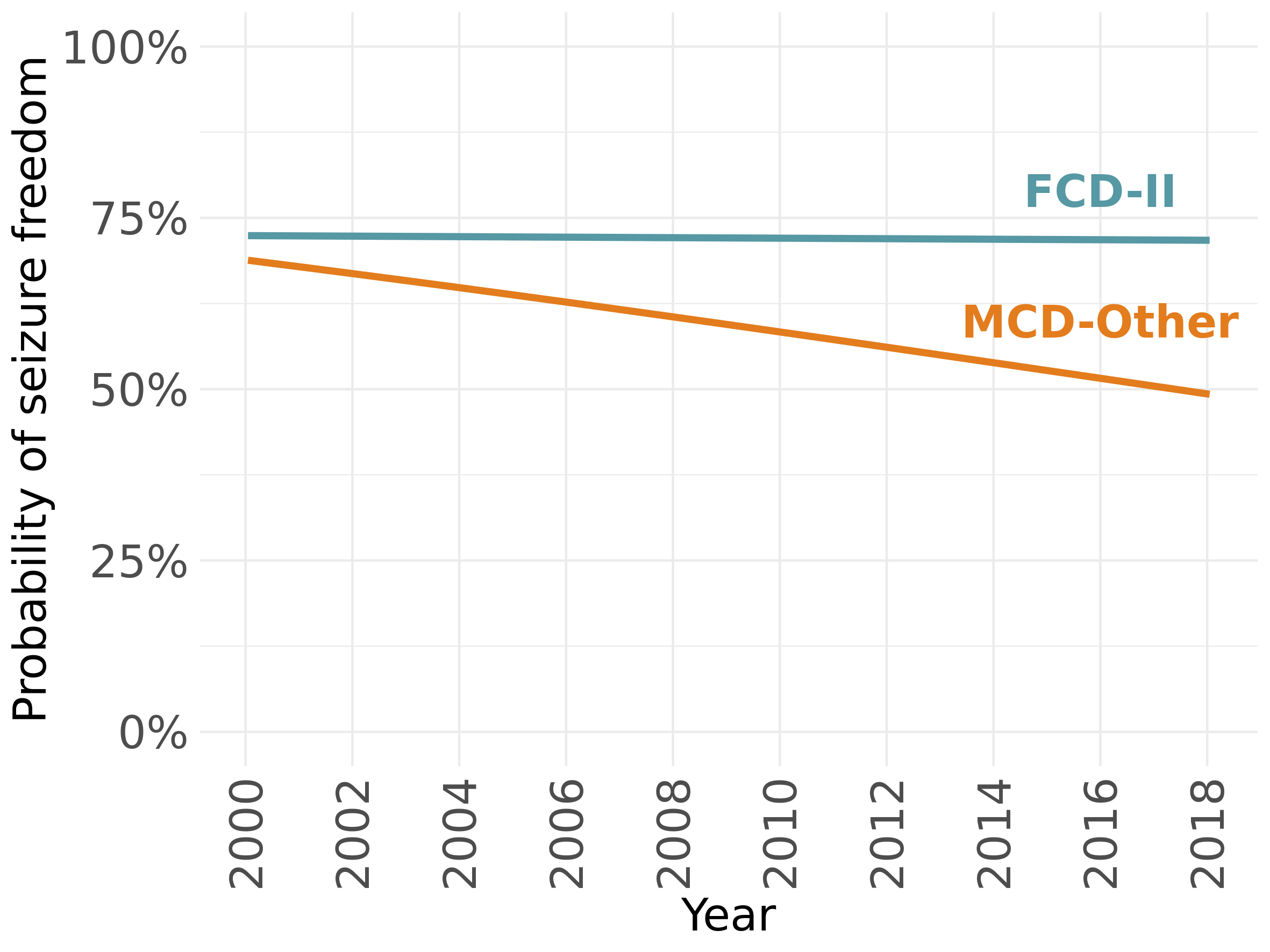


**Supplementary Figure 4.** Patients assigned to the malformation of cortical development-other histopathology category were over time less likely to achieve seizure freedom. Patients with focal cortical dysplasia type II were included in the plot as a comparison, as they showed no change in the likelihood of achieving seizure freedom.


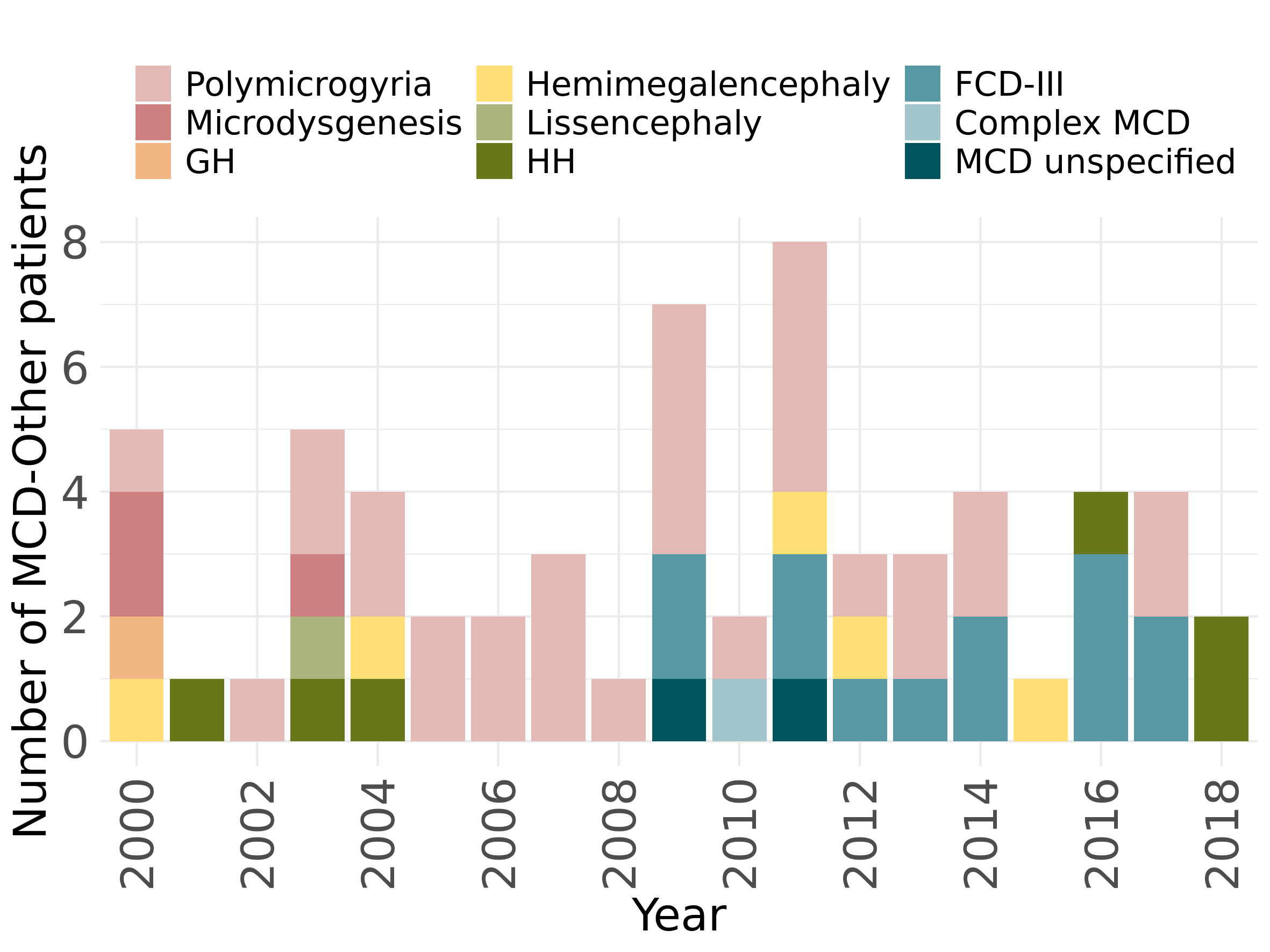


**Supplementary Figure 5.** The malformation of cortical development-other histopathology category was a heterogeneous category, initially comprising predominately of diagnoses of polymicrogyria, but more recently consisting mostly of patients with focal cortical dysplasia type III.

Abbreviations: FCD-II = Focal cortical dysplasia type II; FCD-III = Focal cortical dysplasia type III; GH = Glioneuronal hamartoma; HH = Hypothalamic hamartoma; MCD-Other = Malformation of cortical development-other.

| **Supplementary Table 7. Probability of achieving seizure freedom over time, by pre-operative MRI status.** Multivariable logistic regression with seizure freedom at one-year follow-up as outcome. | | | |
| --- | --- | --- | --- |
| **Variable** | **OR** | **95% CI** | ***p*-Value** |
| Focal MRI abnormality * Procedure date | (ref) |  |  |
| Non-focal MRI abnormality * Procedure date | 1.00 | (0.94, 1.07) | 0.924 |
| MRI negative * Procedure date | 1.08 | (0.92, 1.31) | 0.366 |
| Abbreviations: CI = Confidence interval. OR = Odds ratio. | | | |

| **Supplementary Table 8. Probability of achieving seizure freedom over time, by surgery type.** Multivariable logistic regression with seizure freedom at one-year follow-up as outcome. | | | |
| --- | --- | --- | --- |
| **Variable** | **OR** | **95% CI** | ***p*-Value** |
| Lesionectomy * Procedure date | (ref) |  |  |
| Corpus callosotomy * Procedure date | 1.06 | (0.82, 1.48) | 0.694 |
| Disconnection * Procedure date | 0.98 | (0.83, 1.15) | 0.771 |
| Hemispherotomy * Procedure date | 0.98 | (0.90, 1.07) | 0.657 |
| Lobectomy * Procedure date | 0.97 | (0.90, 1.04) | 0.346 |
| Abbreviations: CI = Confidence interval. OR = Odds ratio. | | | |
